# Evaluation of the vector competence for Batai virus of native *Culex* and exotic *Aedes* species in Central Europe

**DOI:** 10.1186/s13071-024-06296-4

**Published:** 2024-05-15

**Authors:** Anna Heitmann, Magdalena Laura Wehmeyer, Renke Lühken, Konstantin Kliemke, Hanna Jöst, Norbert Becker, Michelle Helms, Jonas Schmidt-Chanasit, Stephanie Jansen

**Affiliations:** 1 Bernhard Nocht Institute for Tropical Medicine, 20359 Hamburg, Germany; 2 Institute for Dipterology (IfD), 67346 Speyer, Germany; 3 Center for Organismal Studies (COS), University of Heidelberg, 69120 Heidelberg, Germany; 4 Faculty of Mathematics, Informatics and Natural Sciences, University of Hamburg, Hamburg, Germany

**Keywords:** BATV, *Culex torrentium*, Vector competence, *Aedes albopictus*, *Aedes japonicus japonicus*, *Culex pipiens pipiens*

## Abstract

**Background:**

Batai virus (BATV) is a zoonotic arbovirus of veterinary importance. A high seroprevalence in cows, sheep and goats and infection in different mosquito species has been observed in Central Europe. Therefore, we studied indigenous as well as exotic species of the genera *Culex* and *Aedes* for BATV vector competence at different fluctuating temperature profiles.

**Methods:**

Field caught *Culex pipiens* biotype *pipiens*, *Culex torrentium*, *Aedes albopictus* and *Aedes japonicus japonicus* from Germany and *Aedes aegypti* laboratory colony were infected with BATV strain 53.3 using artificial blood meals. Engorged mosquitoes were kept under four (*Culex* species) or three (*Aedes* species) fluctuating temperature profiles (18 ± 5 °C, 21 ± 5 °C, 24 ± 5 °C, 27 ± 5 °C) at a humidity of 70% and a dark/light rhythm of 12:12 for 14 days. Transmission was measured by testing the saliva obtained by forced salivation assay for viable BATV particles. Infection rates were analysed by testing whole mosquitoes for BATV RNA by quantitative reverse transcription PCR.

**Results:**

No transmission was detected for *Ae. aegypti*, *Ae. albopictus* or *Ae. japonicus japonicus*. Infection was observed for *Cx. p. pipiens*, but only in the three conditions with the highest temperatures (21 ± 5 °C, 24 ± 5 °C, 27 ± 5 °C). In *Cx. torrentium* infection was measured at all tested temperatures with higher infection rates compared with *Cx. p. pipiens*. Transmission was only detected for *Cx. torrentium* exclusively at the highest temperature of 27 ± 5 °C.

**Conclusions:**

Within the tested mosquito species, only *Cx. torrentium* seems to be able to transmit BATV if the climatic conditions are feasible.

**Graphical Abstract:**

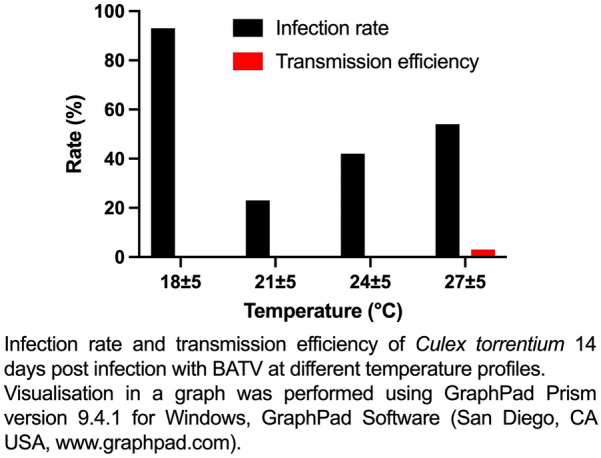

## Background

Batai virus (BATV) [1, 2] belongs to the genus *Orthobunyavirus* within the family *Peribunyaviridae* [3]. Initially detected in *Culex gelidus* trapped in Malaysia in 1955, it has since been identified in southern Slovakia (referred to as Calovo virus, CVOV) [4, 5] as well as in various European countries (for a review, see [6]). Another variant, Chittoor virus (CHITV), has been found in *Anopheles barbirostris* in India [7].

This zoonotic and especially veterinary important virus is transmitted by mosquitoes and biting midges, with mosquitoes considered as the most important vector group [8]. It affects a variety of vertebrate hosts, including pigs, horses, ruminants and various bird species. BATV has been detected in Africa, Europe and Asia. Human infections are rare and associated with mild flu-like symptoms. Infections of pigs, wild birds and harbour seals have been detected, and in ruminants severe outcomes such as abortions, premature births and genetic defects have been noted [1, 9].

The genomic structure of orthobunyaviruses is tripartite consisting of single-stranded RNA genomes [10]. This tripartite genome organisation leads to the appearance of reassortants, most frequently amongst co-circulating, genetically closely related strains [11].

Reassortments within the genus *Orthobunyavirus* may lead to viruses capable of inducing severe symptoms in humans [12]. Ngari virus, which carries the L- and S-segment of Bunyamwera orthobunyavirus and the M-segment of BATV, is associated with increased viral titres in infected mammalian cells as well as increased pathogenicity compared with the parental viruses [13, 14]. Ngari virus has been responsible for at least two outbreaks of haemorrhagic fever in humans in Central Africa between 1998 and 1999 [15, 16].

Surveillance studies conducted in Germany and Italy have confirmed the presence of antibodies against BATV in cattle, sheep and goats. Overall, these studies have demonstrated a seroprevalence up to 44% [17, 18]. However, in Europe, BATV-associated disease has not yet been reported in ruminants or humans. Notably, a BATV infection has been detected in a German captive harbour seal that exhibited encephalitis symptoms [9].

Furthermore, BATV has been repeatedly detected in Germany *Anopheles maculipennis* s.l., in Germany and twice in Italy [19–21]. Additionally, BATV has been identified in various other taxa, including *Culex pipiens* [22].

Recent laboratory studies with the Asian lineage of BATV showed that the mosquito species *Culex quinquefasciatus* as well as *Culex tritaeniorhynchus* are able to transmit the virus, whereas *Aedes aegypti* could only be infected [23]. British *Cx. pipiens* could also only be infected with BATV, while *Aedes detritus* was a competent vector under laboratory conditions [24].

Taken together, several mosquito species in Central Europe could potentially act as vectors for BATV. We recently showed that especially *Culex torrentium*, one of the three most frequent *Culex* species in Central Europe [*Cx. p. biotype pipiens* (*Cx. p. pipiens*), *Cx. p. biotype molestus* (*Culex p. molestus*) and *Cx. torrentium*] is a potent vector for arboviruses, e.g. West Nile virus (WNV) and Sindbis virus [25, 26]. In addition, the exotic species *Aedes albopictus* has infested more than 20 countries in Europe and is established along the Upper Rhine Valley in Germany and France and is known as a competent vector for chikungunya virus (CHIKV) and dengue virus (DENV) [27–30].

We assessed the vector competence of field-caught *Culex* species *Cx. p. pipiens* and *Cx. torrentium* as well as the invasive species *Ae. albopictus*, *Aedes japonicus japonicus* along with the laboratory colony of *Ae. aegypti* as a reference. Vector competence, in this context, refers the inherent ability of a mosquito to be infected and subsequently transmit the virus [31], confirmed by the presence of infectious viral particles in the mosquito’s saliva. Additionally, we investigated the impact of varying temperatures on the risk of BATV transmission by these different mosquito species.

## Methods

*Culex* egg rafts were collected in Hamburg, Neugraben-Fischbek, Germany (longitude 53.467821/latitude 9.831346) in 2018 and 2019. Egg rafts were individually reared at room temperature with a 12:12 light:dark photoperiod. Molecular identification as *Cx. p. pipiens* and *Cx. torrentium* was performed using DNA extraction of a pool of five L1/L2 larvae per egg raft (DNeasy blood & tissue kit, Qiagen, Hilden, Germany) in a multiplex quantitative real-time PCR (HotStarTaq master mix kit, Qiagen, Hilden, Germany) as described [32].

*Aedes albopictus* were reared from a laboratory colony originally collected from Heidelberg, Germany (F26-29) and *Ae. aegypti* were reared from a historic laboratory colony from the Bayer company (Leverkusen, Germany). *Ae. japonicus* were reared from eggs collected with ovitraps in southwestern Germany (longitude 8.671355/lattitude 49.523888) in summer 2019. All adult mosquitoes were reared at 26 °C, with a relative humidity of 70% and a 12:12 light:dark photoperiod with 30 min twilight.

Females (7–10 days old) were starved for 24 hours (*Aedes*) or 48 hours (*Culex*). The artificial blood feeding was conducted at 24 °C for 2 hours. The blood meal consisted of 50% human blood (expired blood preservation), 30% of an 8% fructose solution, 10% filtrated bovine serum (FBS) and 10% virus stock, and was fed using a cotton stick (*Culex*) or two 50 µl drops (*Aedes*) on the bottom of the vial as previously described [33]. The virus stock contained BATV of the European lineage [strain 53.2, Genbank numbers HQ455790 (S-segment), HQ455791 (M-segment) and HQ457992 (L-segment)] isolated from *An. maculipennis* s.l. collected in Southern Germany [19] at a final concentration of 10^7^ plaque forming units per millilitre (PFU/mL). BATV stock was produced and quantified via TCID50 on Vero cells (*Chlorocebus sabaeus*; CVCL_0059, obtained from ATCC, cat. no. CCL-81), results were converted in PFU/mL and the stock was diluted to reach a final concentration of 10^7^ PFU/mL.

Only fully engorged females were used in the following experiments (ten females per vial). An 8% fructose solution was available via soaked cotton pads over the timeframe of the experiment. In general, mosquitoes were incubated for 14 days at 70% humidity and oscillating temperature profiles with mean temperatures of 18, 21, 24 and 27 °C and variations of ± 5 °C within 24 hours to mimic day–night temperature variations as previously described [29]. A diurnal temperature range of approximately 10 °C is commonly observed in the summer months in Germany [34]. The temperature maximum was reached in the middle of the light period, the temperature minimum in the middle of the dark period. Temperature profiles will be referred to by their mean temperature in the following text.

*Culex* mosquitoes were tested for all four mean temperatures in parallel. *Aedes* mosquitoes were tested at the highest mean temperature and at one lower temperature in parallel (21 °C for *Ae. aegypti*/*Ae. japonicus* and 24 °C for *Ae. albopictus*).

The salivation assay was performed at 14 days post infection (dpi) in alignment with previous studies [28, 29]. In summary, mosquitoes were anaesthetised using CO_2_ to facilitate the removal of legs and wings. The proboscis was then placed into a 10 µL tip containing phosphate-buffered saline (PBS) and incubated for 30 min. To test for viable virus particles, each saliva/PBS mix was incubated on Vero cells seeded in a 96-well plate for 7 days. To confirm the presence of BATV RNA, supernatant of Vero cells showing cytopathic effect were prepared for additional RNA testing as recently described by Jansen et al. [29]. RNA was isolated using the QIAamp Viral RNA Mini kit (Qiagen, Hilden, Germany). BATV RNA was detected using the quantitative real-time RT–PCR (qRT–PCR) as previously described [19] using the primers BATAI-Fwd (5′-GCTGGAAGGTTACTGTATTTAATAC-3′) and BATAI-Rev (5′-CAAGGAATCCACTGAGTCTGTG-3′) and the probe BATAI-P (5′-FAM-AACAGTCCAGTTCCAGACGATGGTC-BHQ). A series of a synthetic BATV (1.15 × 10^3^, 1.15 × 10^4^ and 1.15 × 10^5^ copies) standards spanning the qRT–PCR product with an additional 5′ GTA and 3′ ACG overhang (5′-GTAGCTGGAAGGTTACTGTATTTAATACCGTAACAGTCCAGTTCCAGACGATGGTCAGTCACAGACTCAGTGGATTCCTTGACG-3′) was used as a positive control and for quantification of RNA copies within the sample, the threshold for positive PCR results was 100 copies per mosquito.

Every mosquito excluding legs and wings was homogenised using a micro homogeniser (Thermo Fisher Scientific, Waltham, Massachusetts, USA) in 500 µL Dulbecco’s modified Eagle medium (DMEM) and RNA was isolated using the 5× MagMax Pathogen RNA/DNA kit (Thermo Fisher Scientific, Waltham, Massachusetts, USA) as indicated in the manual. BATV RNA was detected via qRT–PCR as mentioned above. The mean number of RNA copies per mosquito was determined per temperature and species (log10 BATV RNA copies/mosquito).

We determined the feeding rate (FR, the number of engorged mosquitoes per number of mosquitoes that were offered an infectious blood meal) infection rate (IR, number of viral RNA positive mosquitoes per number of engorged mosquitoes), transmission rate (TR, the number of mosquitoes with BATV positive saliva per number of viral RNA positive mosquito bodies), transmission efficiency (TE, the number of mosquitoes with BATV positive saliva/number of engorged mosquitoes) and survival rate (SR, number of surviving mosquitoes on day 14 per number of engorged mosquitoes).

## Results

For *Ae. aegypti*, infection rates of 40% at 21 °C and 29% at 27 °C were detected, the mean number of copies ranged between 4.1 and 4.2 log10 RNA copies/mosquito. Transmission could not be detected (Table 1).

**Table 1:** Results of vector competence studies with BATV for tested *Aedes* species

Species	FR (%)	Temperature (°C)	*n*	IR (%) [*]	Mean number of RNA copies per mosquito( log10 BATV RNA copies/mosquito) (95% confidence interval)	TR (%) [**]	TE (%) [***]	SR (%)
*Ae. aegypti*	71	21 ± 5	35	40 [14/35]	4.1 [3.7–4.4]	0 [0/14]	0 [0/35]	85
		27 ± 5	35	29 [10/35]	4.2 [3.9–4.5]	0 [0/10]	0 [0/35]	87
*Ae. albopictus*	58	24 ± 5	60	3 [2/60]	7.6 [3.5–11.7]	0 [0/2]	0 [0/60]	85
		27 ± 5	60	12 [7/60]	4.8 [4.3–4.8]	0 [0/7]	0 [0/60]	N/A
*Ae.japonicus*	71	21 ± 5	7	86 [6/7]	5,99 [5.6–6.4]	0 [0/6]	0 [0/7]	N/A
		27 ± 5	6	50 [3/6]	5,03 [5–5.1]	0 [0/6]	0 [0/6]	N/A

Feeding rates (FR), infection rates (IR), mean number of RNA copies per mosquito, transmission rate (TR); transmission efficiency (TE) and survival rates (SR) of *Ae. aegypti*, *Ae. albopictus* and *Ae. japonicus* 14 days post infection (dpi) at different temperatures

FR: number of engorged mosquitoes per number of mosquitoes that were offered an infectious blood meal; IR: number of positive mosquitoes per number of engorged mosquitoes [*]; TR: number of mosquitoes with positive saliva per number of positive mosquitoes [**]; TE: number of mosquitoes with positive saliva/number of engorged mosquitoes [***]; SR: surviving mosquitoes on day 14 per number of engorged mosquitoes; N/A: not analysed (data missing); *n*: number of engorged mosquitoes

*Aedes albopictus* females were only infected at the two higher temperatures of 27 °C and 24 °C. A rather low infection rate of 3.3% was detected at 24 °C (Table 1). At 27 °C the infection rate was slightly higher with 11.7%. Mean numbers of RNA copies per mosquito ranged between 4.8 and 7.6 log10 RNA copies/mosquito. Transmission could not be detected at either of the investigated temperatures.

For *Ae. japonicus*, infection but no transmission could be shown at the tested temperature of 27 °C (IR of 50%) and 21 °C (IR of 86%) (Table 1). Mean number of RNA copies per mosquito ranged between 5.03 and 5.99 log10 RNA copies/mosquito.

*Culex p. pipiens* females could be infected with BATV after incubation at 21, 24 and 27 °C, with infection rates between 8.1% and 50% (Table 2).

**Table 2:** Results of vector competence studies with BATV tested *Culex* species

Species	FR (%)	Temperature (°C)	*n*	IR (%) [*]	Mean number of RNA copies per mosquito( log10 BATV RNA copies/mosquito) (95% confidence interval)	TR (%) [**]	TE (%) [***]	SR (%)
*Cx. pipiens* biotype *pipiens*	46	18 ± 5	30	0 [0/30]	/	0 [0/0]	0 [0/30]	84
		21 ± 5	30	50 [15/30]	5.5 [4.6–6.4]	0 [0/15]	0 [0/30]	92
		24 ± 5	33	10 [3/30]	6.0 [5.3–6.6]	0 [0/3]	0 [0/30]	90
		27 ± 5	33	27.3 [9/30]	5.1 [4.8–5.5]	0 [0/9]	0 [0/30]	88
*Cx. torrentium*	54	18 ± 5	30	93.3 [28/30]	4.6 [4.3–4.9]	0 [0/28]	0 [0/30]	79
		21 ± 5	31	22.6 [7/31]	5.0 [4.4–5.5]	0 [0/31]	0 [0/30]	95
		24 ± 5	33	42.4 [14/33]	5.4 [4.8–5.9]	0 [0/14]	0 [0/30]	100
		27 ± 5	33	54.55 [18/33]	6.0 [4.5–7.1]	5.5 [1/18]	3 [1/33]	100

Feeding rates (FR), infection rates (IR), mean number of RNA copies per mosquito, transmission rate (TR), transmission efficiency (TE) and survival rates (SR) of *Cx. p. pipiens* and *Cx. torrentium* 14 days post infection (dpi) at different temperatures

FR: number of engorged mosquitoes per number of mosquitoes that were offered an infectious blood meal; IR: number of positive mosquitoes per number of engorged mosquitoes [*]; TR: number of mosquitoes with positive saliva per number of positive mosquitoes [**]; TE: number of mosquitoes with positive saliva/number of engorged mosquitoes [***]; SR: surviving mosquitoes on day 14 per number of engorged mosquitoes; N/A: not analysed (data missing); *n*: number of engorged mosquitoes

For *Culex*, no specific temperature effect was detected for the three higher temperatures, while no infection could be detected at the lowest temperature of 18 ± 5 °C. Mean number of RNA copies per mosquito ranged between 5.1 and 6.0 log10 RNA copies/mosquito. Transmission could not be detected for *Cx. p. pipiens*. *Culex torrentium* showed infection at all temperatures, there was no hint towards a temperature dependency concerning the infection. Overall infection rates were higher compared with *Cx. p. pipiens,* with values between 22.6% and 93.3% (Table 2). *C. torrentium* was also able to transmit the virus at the highest of the tested temperatures with a low transmission efficiency of 3%. At this temperature, mean number of RNA copies per mosquito reached the highest values of 6.0 log10 RNA copies/mosquito, in comparison with 4.6–5.4 log10 RNA copies/mosquito at the other temperatures (Table 2).

In addition, we measured the survival of *Cx. p. pipiens*, *Cx. torrentium*, *Ae. aegypti* and *Ae. albopictus* (only at 24 °C) at 14 days after infection. Independent of the incubation conditions or the tested mosquito species, survival rates never fell below 79%. For *Cx. torrentium* even survival rates of 100% were detected at the highest temperatures.

## Discussion

The presence of BATV antibodies has been studied in Eastern Germany across various livestock species including sheep, goat and cattle [17, 18, 35]. These studies have revealed seroprevalences as high as 44.7%. Antibodies have also been detected in bovine serum samples from the Novarra region in Northern Italy in 2011 [36]. Furthermore, BATV RNA has been detected in aedine and culicine mosquitoes in Germany [19, 22] and in a pool of *An. maculipennis* s.l. mosquitoes in Italy [21]. These findings collectively suggest that the virus is circulating in Central Europe, particularly in regions such as Eastern Germany. Despite the absence of documented BATV infections in humans, a BATV infection has been detected in Germany in a captured harbour seal showing symptoms of encephalitis [9].

However, no further documented BATV infections have been reported in humans or livestock in Central Europe. Despite this, it remains crucial to continue investigating BATV, as the overall risk of arbovirus transmission is on the rise.

In recent years, the risk of introduction and establishment of arboviral transmission cycles within Central Europe has grown. Notable examples are CHIKV epidemics in Italy and dengue virus (DENV) case clusters in Spain, France and Italy have been described [37, 38]. These outbreaks are attributed to factors such as the expanding distribution of the known CHIKV and DENV vector *Ae. albopictus*, as well as rising temperatures. Furthermore, there is also circulation of endemic viruses such as WNV. It emerged in Germany in 2018, and caused epidemics in Greece and Italy since 2010 [39, 40] with higher temperatures being one of the driving factors [25].

Specifically, BATV could pose a threat, parallels can be drawn from Cache Valley virus (CVV) another member of the Bunyamwera serogroup. In small ruminants, CVV infection may lead to foetal death or severe malformation of the foetus [41]. Cache Valley virus circulates in North, Central and South America and has been isolated from over 40 mosquito species [41]. Although human cases are rare, symptoms can range from mild illness with fever to severe cases of encephalitis. Notably, recent studies in the USA have revealed an increase in CVV infections. They showed that the invasive species *Ae. albopictus* transmits this virus and that *Ae. albopictus* is widespread in the area where CVV cases have been detected [42]. Based on these findings, we conducted tests on *Aedes* species, particularly the invasive ones, to assess their potential impact on the transmission of BATV in Central Europe.

Furthermore, reassortments within the Bunyamwera serogroup occur naturally. The most notable example is a reassortment event between BATV and Bunyamwera virus, resulting in the emergence of Ngari virus. Reassortment has led to an increase in pathogenicity, contributing to two major haemorrhagic fever outbreaks in humans in Africa [15]. Given their close genetic relationship, knowledge about competent vectors for BATV could inform risk assessments related to Ngari virus outbreaks.

As observed before, the laboratory colony of *Ae. aegypti* could be infected with BATV at both of the tested temperatures (27 °C, 21 °C), which were chosen to reflect a tropical and a more moderate temperature. No transmission could be detected, which is in line with previous studies [22]. For the other studied invasive *Aedes* species (*Ae. albopictus* and *Ae. japonicus*) similar results were observed at the two tested temperature, being 27 °C and 24 °C for *Ae. albopictus* and 27 °C and 21 °C for *Ae. japonicus* (infection but no transmission). None of the tested *Aedes* species were competent vectors. However, the sample size of *Ae. japonicus* was smaller than that of the other tested species. With six tested mosquitoes, the minimal detection limit is a TE of 16%, but TEs below this might already be biological relevant, therefore *Ae. japonicus* could still be competent vector here defined as the presence of viable virus particles within the saliva. The number of at least 30 investigated specimens per condition is well established in the field of vector competence studies and allows to determine TEs as low as 3%. Biologically relevant vector competence can be determined (TE > 3%), but the effort of the experiments is still proportionate.

Vector competence studies with *Ae. albopictus* and CVV already revealed that different lineages of CVV have a remarkable effect on transmission [42]. Although no transmission of BATV by *Ae. albopictus* was detected in this study, *Ae. albopictus* still could possibly contribute to the transmission of other strains of BATV if they would be introduced. Therefore, it would be of interest to test *Ae. albopictus* and other *Aedes* species for different BATV strains.

No obvious effect of BATV infection on survival could be seen for any of the tested species. This includes *Cx. torrentium* the only species that tested positive for BATV in the saliva in this study. This is in line with recently published results, where negative effects on survival could only be shown for *Ae. detritus*, but no changes in mortality could be observed for *Ae. aegypti* or *Cx. pipiens*. [24].

As BATV is transmitted by over 40 [41] different species from different genera, we included additional information regarding two specific species: *Cx. p. pipiens* and *Cx. torrentium*. These species are most abundant *Culex* species in Europe and previous research has demonstrated that these two serve as potential bridge vectors [43]. Recently, it has been shown for the Asian lineage of BATV, that *Cx. quinquefasciatus* as well as *Cx. tritaeniorhynchus* are competent vectors [23]. In contrast, it has been shown that a *Cx. pipiens* laboratory colony (hybrids from *Cx. p. pipiens* and *Cx. p. molestus*) was not able to transmit the European variant of BATV [24]. Our results for the field-caught *Cx. p. pipiens* are in line with the results obtained for *Cx. pipiens* [24], which were also not able to transmit BATV. However, at the highest temperature of 27 °C, *Cx. torrentium*, the more prevalent species in Central Europe [44] is able to transmit the virus, but only with a low transmission efficiency of 3%. Our data for *Cx. torrentium* show that highest copy numbers in mosquito bodies are reached at the highest temperature.

It has been described that at 20 °C, the extrinsic incubation period of BATV is at least not longer than 7 days in *Ae. detritus* and moreover this study showed that the transmission rate is higher at 7 days compared with 14 days post infection [24]. Therefore, it would be very important to further analyse whether this is also the case for *Cx. torrentium*. To be an effective vector in nature, vector capacity – rather than just vector competence – plays an important role. Vector capacity encompasses physiological, ecological and environmental factors related to the vector, host and pathogen. Key factors include blood-feeding behaviour, temperature and abundances [31]. However, currently neither *Cx. torrentium* nor *Ae. detritus* seems to be the relevant vector responsible for the high seroprevalence detected in several surveillance studies in Eastern Germany [17, 18]. *Ae. detritus* is a halophilic species predominantly distributed in coastal areas [45] and not in the regions described in the studies [17, 18] and *Cx. torrentium* only transmits BATV at high temperatures with a TE of only 3%.

## Conclusion

Within this study, *Cx. torrentium* was found to be a potential vector for BATV at high temperatures but with a low TE. To﻿ unravel the current infection cycle, more mosquito species need to be analysed for their vector competence if technically possible. BATV, for example, has been detected in Germany in a pool of *An. maculipennis* s.l. [19] and in pools of different mosquitoes also containing different *Anopheles* species as well as *Ae. vexans* [21]. Due to their host feeding patterns *Ae. vexans* are important vectors for the transmission from non-human mammals to humans [43]. Combined with the mass appearance of species upon flooding events, they could be an important vector and therefore would be an interesting species to test whether mosquitoes from the field are available. The same is true for *An. maculipennis* s.l.

## Data Availability

All data generated by this study and used is presented within this published article.
